# Transcatheter Proximal Coil Blocking with n-Butyl-2-Cyanoacrylate Injection via the Pulmonary Artery Alone for Rasmussen's Aneurysm

**DOI:** 10.1155/2019/1725238

**Published:** 2019-04-15

**Authors:** Atsushi Ugajin, Hiroyuki Fujii, Hiroyasu Nakamura, Akifumi Fujita, Takahiro Sasaki, Naoko Mato, Hideharu Sugimoto

**Affiliations:** ^1^Department of Radiology, Jichi Medical University, School of Medicine, 3311-1 Yakushiji, Shimotsuke, Tochigi 329-0498, Japan; ^2^Division of Pulmonary Medicine, Department of Internal Medicine, Jichi Medical University, 3311-1 Yakushiji, Shimotsuke, Tochigi 329-0498, Japan

## Abstract

Rasmussen's aneurysm is a peripheral pulmonary artery pseudoaneurysm (PAP) within a tuberculosis cavity. Because it can be perfused from the bronchial and pulmonary arterial circulations, combined embolization via the bronchial and pulmonary arteries is sometimes required. Herein, we present case of a 51-year-old man with Rasmussen's aneurysm that was successfully treated by proximal coil blocking with n-butyl-2-cyanoacrylate (NBCA) injection via the pulmonary artery alone. With proximal coil blocking, a sufficient amount of NBCA could be injected without unintended reflux of the NBCA cast to the proximal pulmonary artery. To our knowledge, there has been no report that attempted NBCA injection under proximal coil blocking for Rasmussen's aneurysm. Our treatment approach may be safe and effective for infectious lung disease-related PAP, which has to be treated from the pulmonary artery side.

## 1. Introduction

Tuberculosis is not a disease of the past and remains a serious threat. Hemoptysis in patients with pulmonary tuberculosis can result from various etiologies, including bronchiolitis, bronchiectasis, aspergilloma, or vascular complications. For such cases, bronchial artery embolization (BAE) has become the standard treatment because the hemoptysis usually originates from the bronchial artery or, less frequently, from a nonbronchial systemic artery [1].

On the other hand, Rasmussen's aneurysm, which is a peripheral pulmonary artery pseudoaneurysm (PAP) within a tuberculosis cavity, will sometimes require combined embolization via the bronchial and pulmonary arteries. This is because infectious lung disease-related PAP can be perfused through the bronchial and pulmonary arterial circulations, resulting in recurrent hemoptysis despite BAE [2, 3]. Herein, we present a case of Rasmussen's aneurysm that was successfully treated by proximal coil blocking with n-butyl-2-cyanoacrylate (NBCA) injection via the pulmonary artery alone.

## 2. Case Report

A 51-year-old man was referred to our hospital for treatment of hemoptysis. One week before, he presented with coughing up of approximately 1 cup of bright red blood, which was diagnosed as active pulmonary tuberculosis. He had poorly controlled diabetes mellitus.

**Figure 1:**
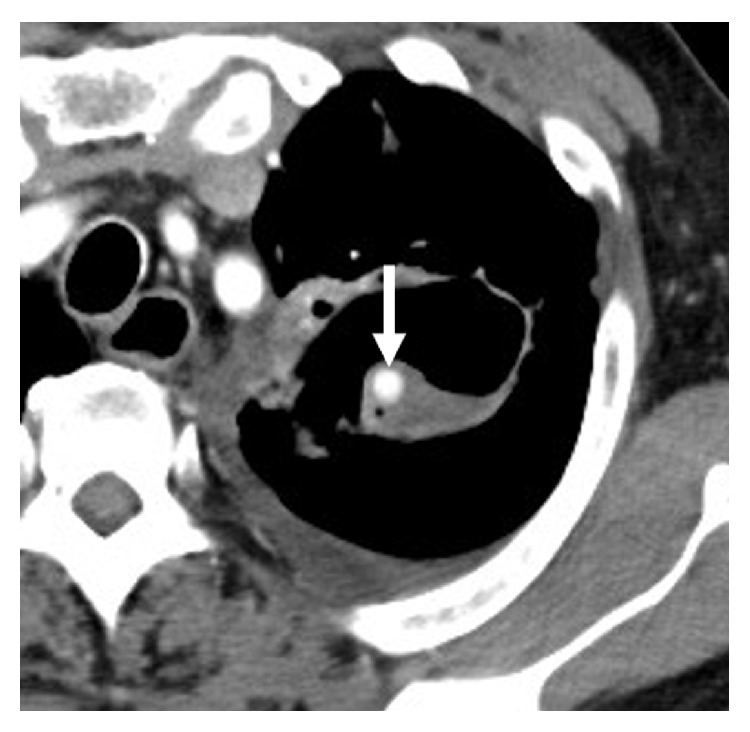
Contrast-enhanced axial computed tomography image. There is a 7 mm round aneurysm (arrow) within a cavitary lesion in the left upper lobe of the lung.

**Figure 2:**
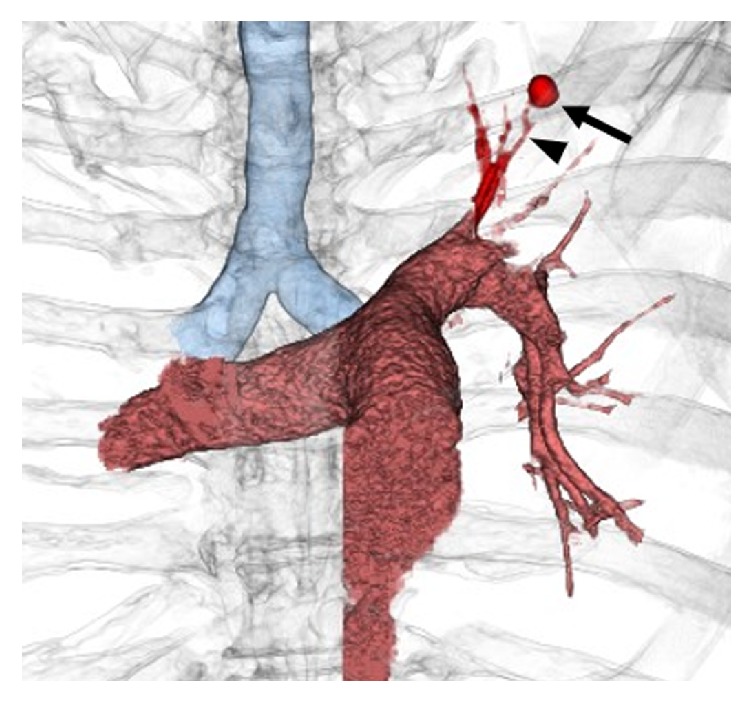
Volume rendering reconstructed image of the computed tomography angiography. The aneurysm (arrow) seems to originate from a branch of the left superior segmental pulmonary artery (arrowhead).

When he arrived at our hospital, hemodynamic status was stable, and there were no abnormal signs or symptoms, except for low-grade fever and mildly elevated C-reactive protein. Contrast-enhanced computed tomography (CT) revealed a 7 mm round pseudoaneurysm within a cavitary lesion in the left upper lobe of the lung (Figure 1). The pseudoaneurysm was thought to originate from a branch of the left superior segmental pulmonary artery (Figure 2). He was diagnosed with Rasmussen's aneurysm and underwent interventional treatment.

**Figure 3:**
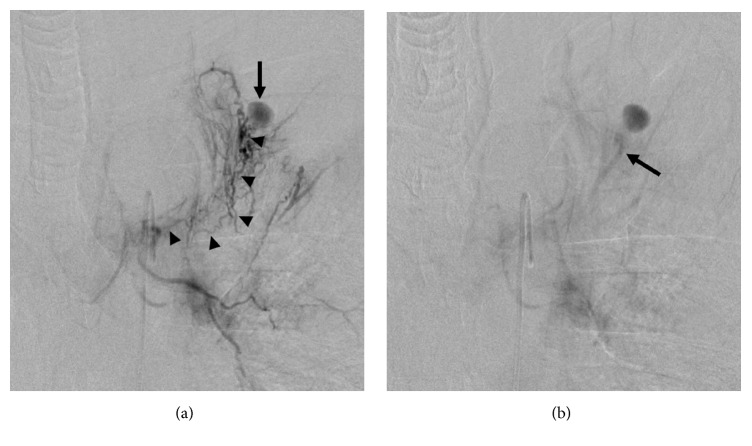
Bronchial angiography. (a) The early phase depicts the aneurysm (arrow) via small and tortuous anastomoses (arrowheads) from the left bronchial artery. (b) The delayed phase shows the parent pulmonary artery of the aneurysm (arrow) via a shunt from the left bronchial artery.

A 5-Fr catheter (SHK, Terumo Clinical Supply, Gifu, Japan) was advanced to the left bronchial artery. A left bronchial angiography depicted the aneurysm via a shunt from the bronchial to the pulmonary artery. However, we could not advance the microcatheter to a more peripheral branch near the lesion, because the anastomosis was small and tortuous (Figure 3). Eventually, subintimal dissection developed in the left bronchial artery and we failed to embolize the aneurysm via the left bronchial artery.

**Figure 4:**
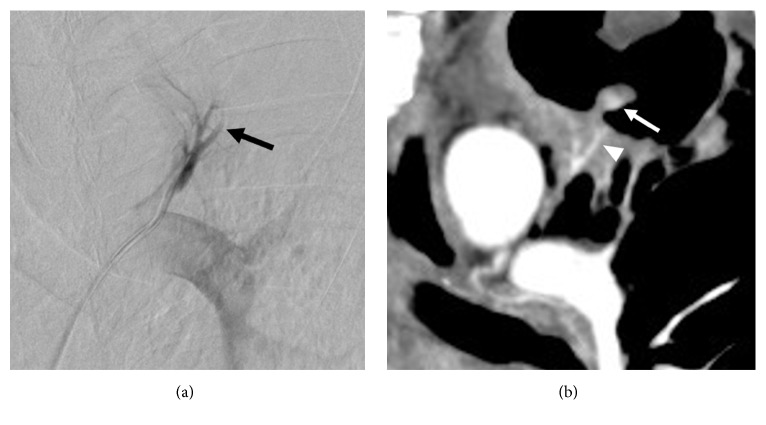
Comparison between pulmonary angiography and computed tomography image. (a) Left segmental pulmonary angiography shows abrupt tapering of the arterial branch (arrow), which indicates retrograde flow from the bronchial to the pulmonary artery. (b) Contrast-enhanced coronal computed tomography image shows the aneurysm (arrow) and the parent artery of the aneurysm (arrowhead). The parent artery corresponds to the abrupt tapering of the arterial branch, which was seen in the left segmental pulmonary angiography.

Subsequently, a 5-Fr guiding catheter (Envoy, Codman Neurovascular, Raynham, Massachusetts, USA) was advanced to the left main pulmonary artery. Left main and left superior segmental pulmonary angiography could not depict the aneurysm. However, we noted an abrupt disappearance of the left superior segmental pulmonary artery, which indicated retrograde flow from the bronchial to the pulmonary artery. The tapering was in the branch that was suspected as the parent artery of the aneurysm on CT (Figure 4). Based on these findings, we were able to identify the parent artery and reach the aneurysm using the microcatheter.

**Figure 5:**
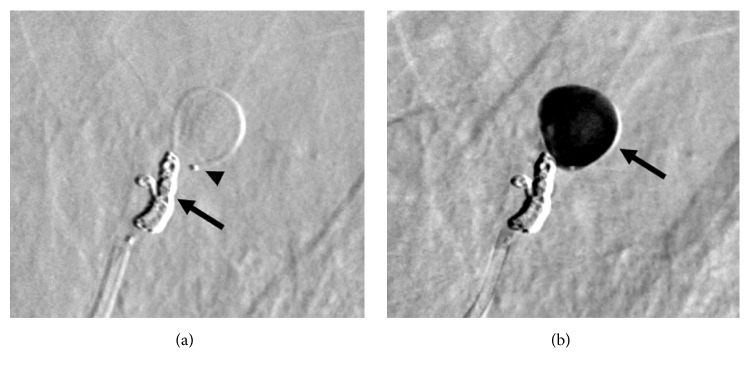
Embolization of the aneurysm. (a) Two microcatheters are placed in the aneurysm and pulmonary artery proximal to the aneurysm. One microcatheter is used for proximal segmental pulmonary artery coil blocking proximal to the aneurysm (arrow); the other microcatheter is positioned into the aneurysm (arrowhead) before injecting a mixture of NBCA and iodized oil. (b) Complete filling of the aneurysm with the mixture of NBCA and iodized oil (arrow) is performed using the other microcatheter. NBCA, n-butyl-2-cyanoacrylate.

**Figure 6:**
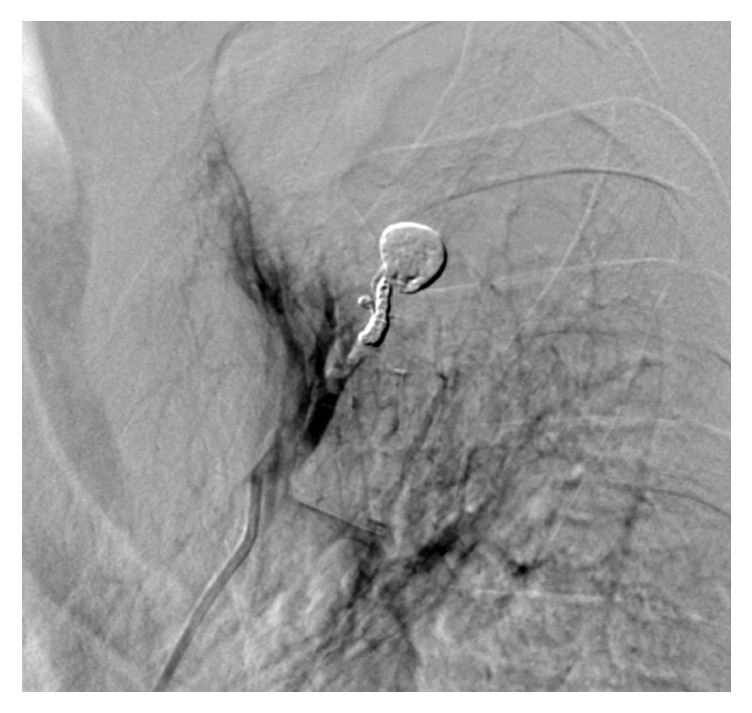
Postembolization assessment. There is no residual aneurysm on pulmonary angiography.

Two microcatheters were placed in the aneurysm and pulmonary artery proximal to the aneurysm. A 1.7-Fr microcatheter (Echelon, ev3, Irvine, California, USA) and a 1.9-Fr microcatheter (Carnelian Marvel, Tokai Medical Products, Aichi, Japan) were inserted in parallel through a guiding catheter. The 1.7-Fr microcatheter was positioned proximal to the aneurysm, whereas the 1.9-Fr microcatheter was advanced into the aneurysm. Because we decided to choose NBCA for embolization, we first performed proximal superior segmental pulmonary artery embolization proximal to the aneurysm using the 1.7-Fr microcatheter, with 3 coils measuring 3 mm × 6 cm, 2.5 mm × 6 cm, and 2 mm × 8 cm (ED COIL10 ExtraSoft Type R, Kaneka Medix, Osaka, Japan) to prevent unintended reflux of the NBCA. Thereafter, a 0.8 mL mixture of NBCA and iodized oil (Lipiodol, Guerbet Japan, Tokyo, Japan) (NBCA:Lipiodol = 1:3) was retrogradely injected into the aneurysm through the remaining 1.9-Fr microcatheter. The aneurysm was filled with the mixture of the NBCA and iodized oil, but the feeding artery could not be embolized retrogradely (Figure 5). Although postembolization bronchial angiography could not be performed due to the subintimal injury in the left bronchial artery, postembolization pulmonary angiography did not show the residual aneurysm (Figure 6).

**Figure 7:**
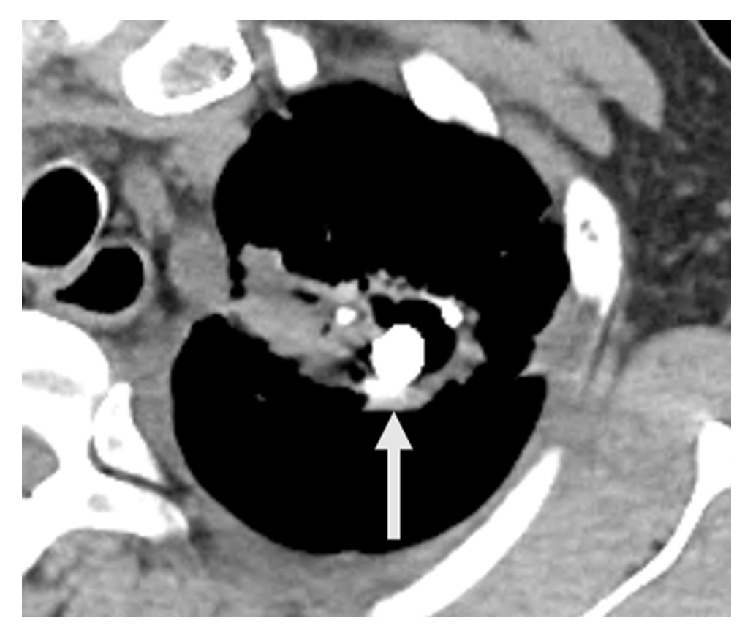
Non-contrast-enhanced axial computed tomography image. Two months after the treatment, the aneurysm is filled with the mixture of n-butyl-2-cyanoacrylate and iodized oil (arrow).

After treatment, he remained stable without further hemoptysis, and there were no other side effects or complications. Follow-up CT performed 2 months later confirmed successful embolization of the aneurysm (Figure 7).

## 3. Discussion

Pulmonary tuberculosis has a significantly lower incidence in developed countries than in developing countries. In developed countries, diabetes mellitus is one of the common risk factors for tuberculosis [4]. The source of the vascular complications underlying massive hemoptysis in tuberculosis is most commonly the bronchial arteries, and only less than 10% is from the pulmonary artery [5, 6]. Rasmussen's aneurysm is a rare complication of pulmonary tuberculosis. A previous report indicated that pseudoaneurysm was present in 4% of patients with tuberculous cavities on autopsy [7]. Rasmussen's aneurysm rupture has a reported incidence of 84% and mortality rate above 80% [7, 8]. Death is usually secondary to aspiration of blood and the consequent asphyxiation. Therefore, it should be treated immediately upon diagnosis.

Endovascular treatment and surgical lobectomy are the treatment options for Rasmussen's aneurysm. Because surgical lobectomy for patients with massive hemoptysis poses a high risk, endovascular treatment has become a widespread initial therapy. However, Rasmussen's aneurysm requires special diagnostic and therapeutic care, owing to its characteristic hemodynamics. The inflammation from infectious lung disease-related PAP can induce bronchial to pulmonary arterial shunt; the flow direction in this shunt is determined by the pressure gradient from the bronchial to the pulmonary artery [9]. The resulting hypoperfusion in the diseased pulmonary segment can affect visualization of the aneurysm on pulmonary angiography [10].

Shin et al. classified infectious lung disease-related PAPs into 4 types, based on bronchial and pulmonary angiographic findings. Type A PAPs can be visualized on nonselective pulmonary angiography, whereas type B PAPs can be visualized on selective pulmonary angiography only. Type C PAPs can be depicted on bronchial and nonbronchial systemic collateral arterial angiography through a bronchial to pulmonary arterial shunt, without visualization of the feeding pulmonary arteries on selective pulmonary angiography. Type D PAPs can be depicted only by pulmonary CT angiography and not by catheter-directed angiography [11]. The present case was categorized as type C Rasmussen's aneurysm.

Recently, multidetector row CT angiography has become an essential diagnostic method before endovascular treatment of hemoptysis of pulmonary artery origin. It can detect the aneurysm and the parent artery, regardless of the hemodynamic pattern [12]. When Rasmussen's aneurysm is detected, 2 embolization methods are suggested to isolate it from perfusion. One method entails BAE followed by selective pulmonary artery embolization, in case a pulmonary angiography shows residual aneurysm. The other method entails selective pulmonary artery embolization followed by BAE, when a residual aneurysm is detected on bronchial angiography. Because BAE has been a popular method for the control of hemoptysis, operators may readily embrace the former method, especially for type C Rasmussen's aneurysm. However, in some cases of Rasmussen's aneurysm, the bronchial artery is tortuous with interconnecting branches, making it difficult to approach the aneurysm via the network-like component. For such cases, the approach may be easy with the use of the second method, because the pulmonary artery is usually short and straight [13].

Several embolic materials, such as gelatin sponge, coils, and NBCA, had been shown to be effective treatment options [11, 13]. Among these, NBCA may be the most feasible material to use, because (1) its liquid form in an appropriate concentration enables embolization of both inflow and outflow arteries, including the aneurysm, and (2) the risk of rupture during filling of the aneurysmal sac may be less, compared with that using coils. However, NBCA is a highly operator-dependent material to use and its behavior is difficult to estimate, especially its retrograde flow from the bronchial to the pulmonary artery. Consequently, nontarget embolization can pose dangerous and serious lung ischemic complications.

**Figure 8:**
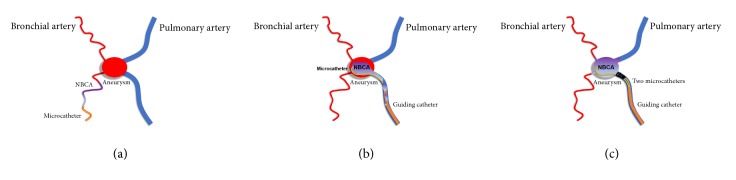
Schematic diagrams of the different approaches to Rasmussen's aneurysm embolization. (a) Via the bronchial artery, only proximal embolization may be achieved because the inflow artery is small and tortuous. (b) Using the conventional approach via the pulmonary artery, selective catheterization of the aneurysm may be easy, but an unintended reflux of the NBCA cast and subsequent incomplete embolization of the aneurysm may occur with the retrograde flow. (c) Using our approach, proximal coil blocking via the pulmonary artery prevents unintended reflux of the NBCA cast and allows continuous retrograde retention of the NBCA in the aneurysm, as well as in the inflow and outflow arteries. NBCA, n-butyl-2-cyanoacrylate.

In the present case, we embolized the aneurysm via the pulmonary artery alone using NBCA injection under proximal segmental pulmonary artery coil blocking. Our approach was able to overcome the above-mentioned difficulties (Figure 8). Proximal coil blocking enables the operator to inject a sufficient amount of NBCA in a retrograde fashion, without the unintended reflux of the NBCA cast to the proximal pulmonary artery. As a recent report pointed out, if pulmonary artery embolization results in incomplete occlusion, additional BAE should be considered to isolate the aneurysm from perfusion [13]. Our treatment may not have been enough, because the feeding artery could not be embolized retrogradely, which meant the possibility of residual inflow to the aneurysm via the bronchial artery. Moreover, bronchial arteriogram after embolization could not be performed due to the subintimal injury in the left bronchial artery. Nevertheless, our treatment method may not require additional BAE, when retrograde injection to the bronchial artery side can be accomplished via the pulmonary artery alone. A similar approach of using microballoon catheter, instead of coil occlusion, for Rasmussen's aneurysm embolization was reported [14]. Our approach negated a concern about adhesion of the refluxed NBCA cast to the inflated balloon, although it carried the risk of coil migration when the glued catheter was pulled out. Another advantage of our approach is that it enables occlusion of a pulmonary artery that has a diameter that is too large for microballooning with more familiar devices, such as coils. To our knowledge, there had been no report that attempted to inject NBCA under proximal coil blocking for Rasmussen's aneurysm.

In conclusion, we presented a case of Rasmussen's aneurysm that was successfully treated via the pulmonary artery alone, using NBCA injection under proximal coil blocking. Our treatment approach may be safe and effective for other cases of infectious lung disease-related PAP that needs to be treated from the pulmonary artery side.
